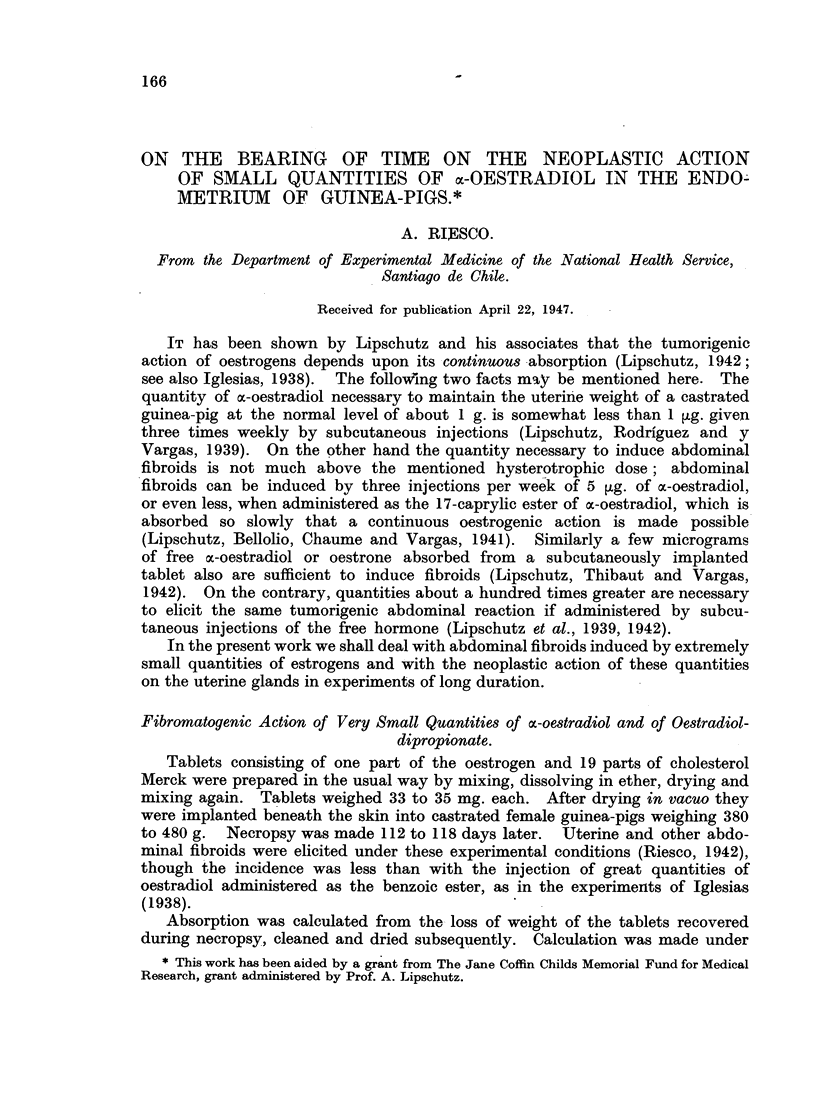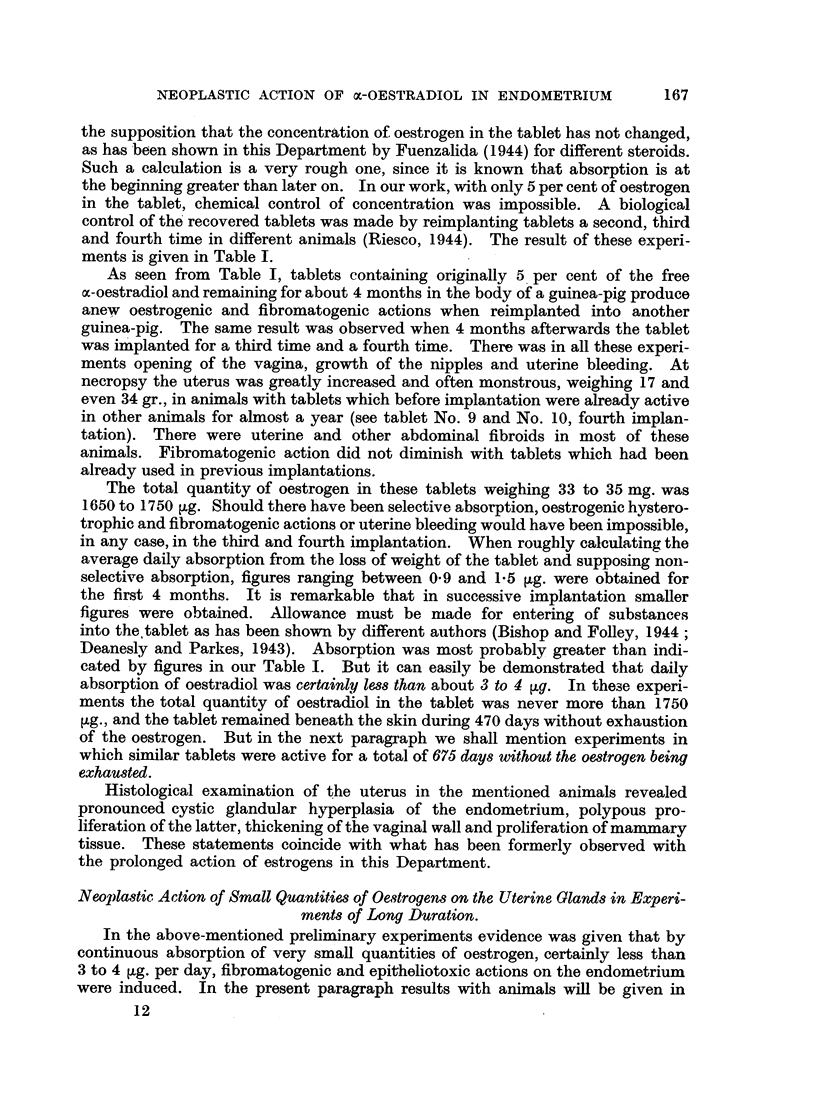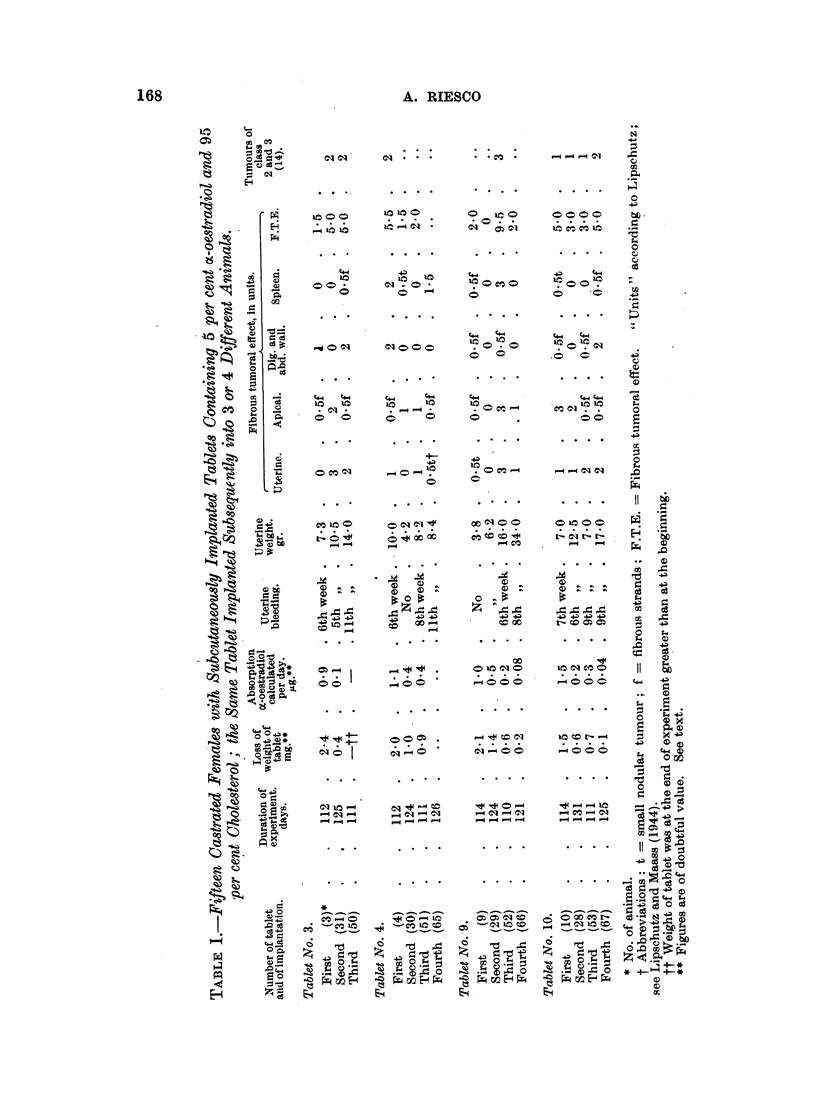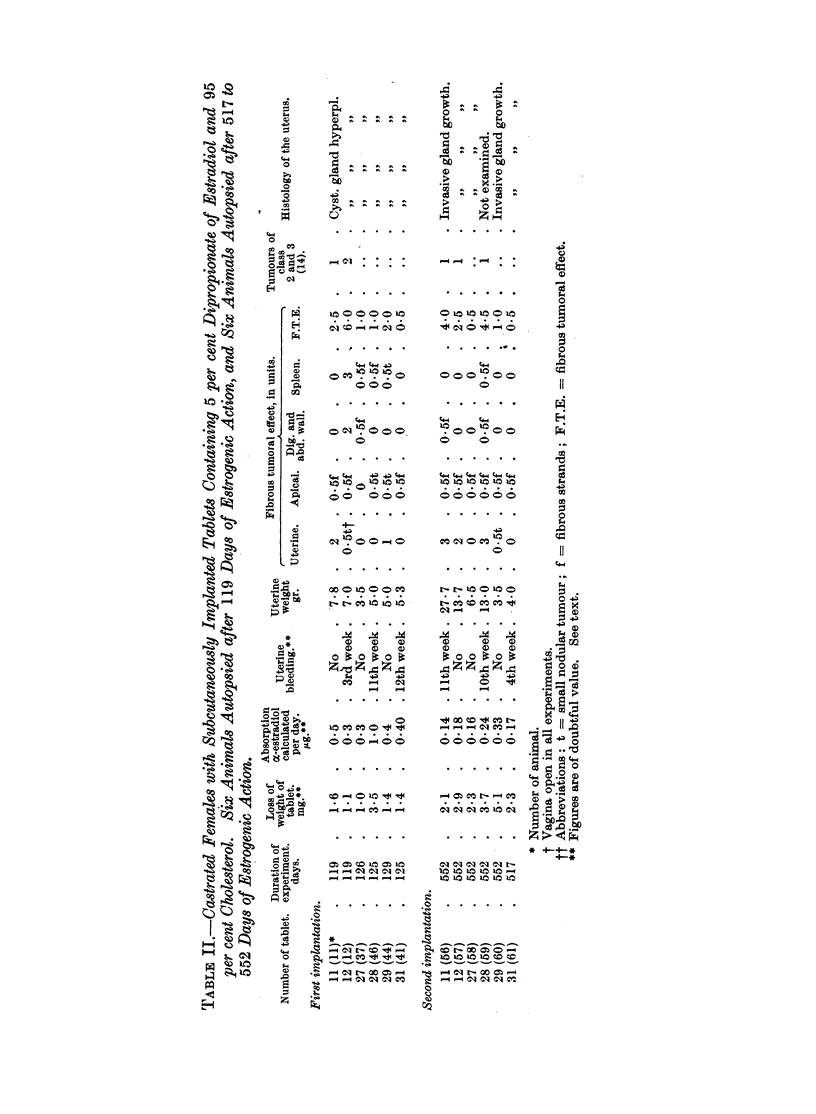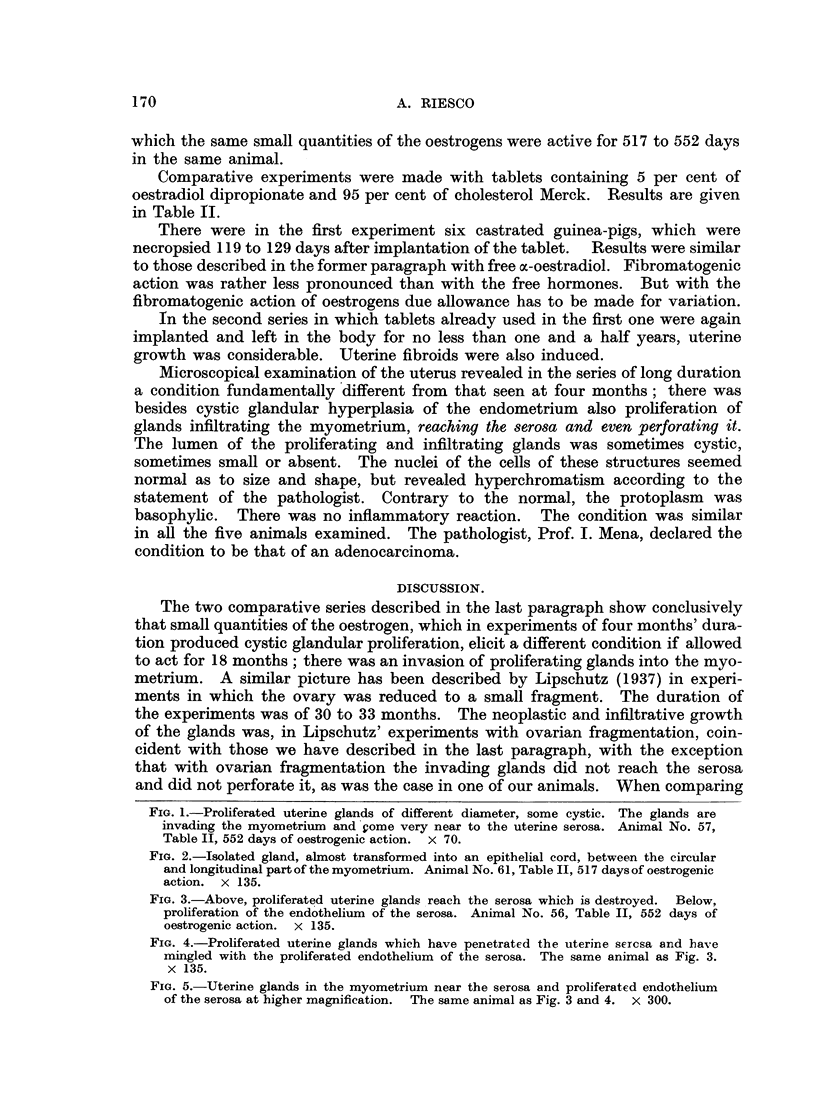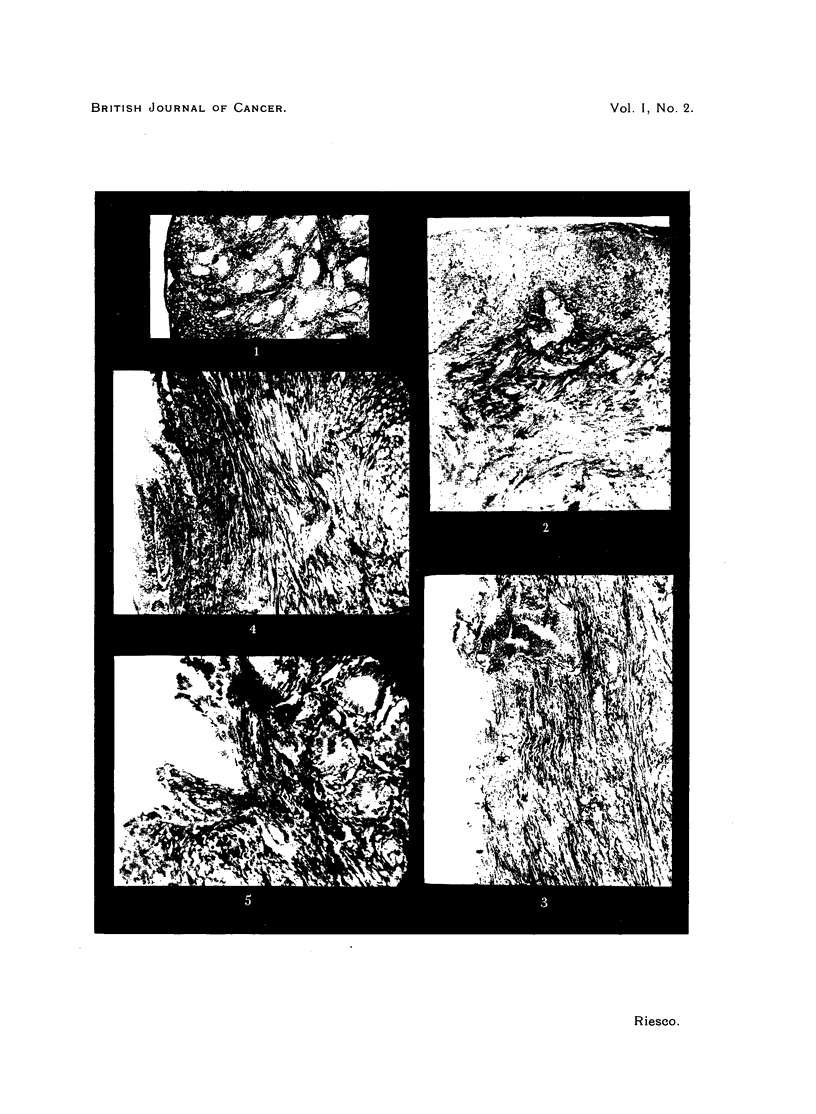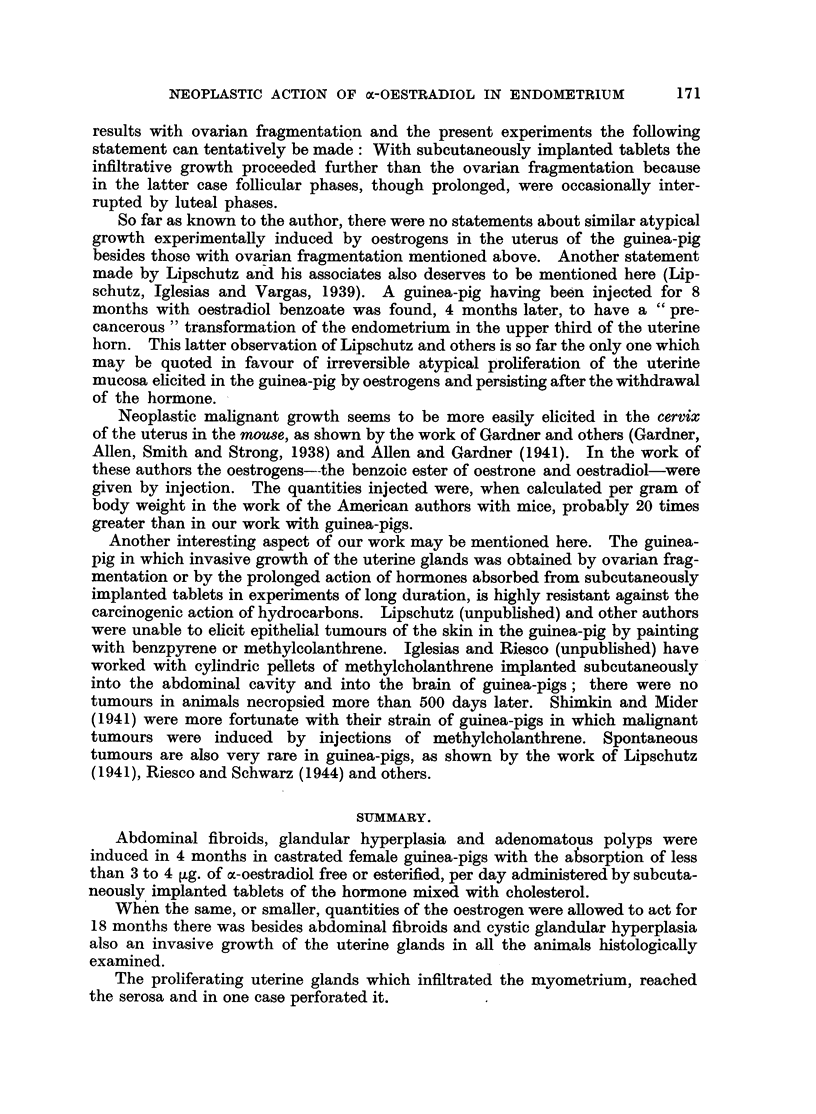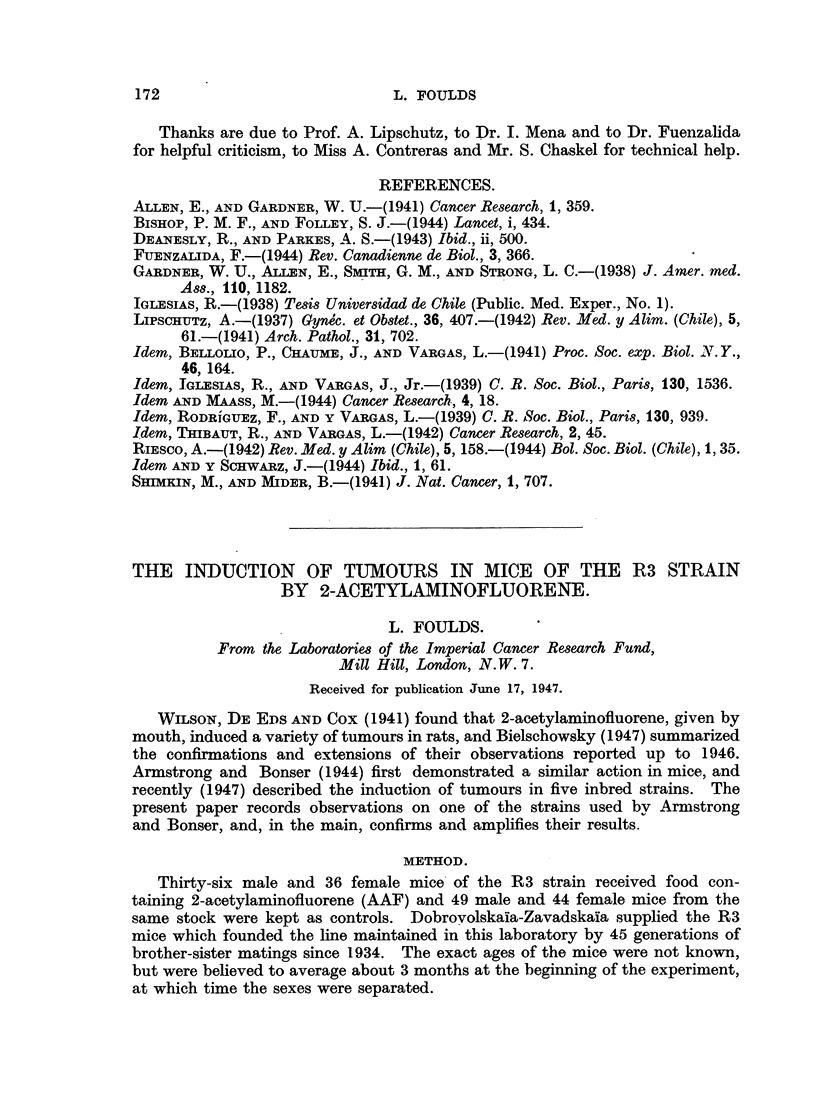# On the Bearing of Time on the Neoplastic Action of Small Quantities of α-Oestradiol in the Endometrium of Guinea-pigs*

**DOI:** 10.1038/bjc.1947.19

**Published:** 1947-06

**Authors:** A. Riesco

## Abstract

**Images:**


### ON THE BEARING OF TIME ON THE NEOPLASTIC ACTION OF SMALL QUANTITIES OF α-OESTRADIOL IN THE ENDOMETRIUM OF GUINEA-PIGS.*

A. RIESCO.

*From the Department of Experimental Medicine of the National Health Service, Santiago de Chile.*

Received for publication April 22, 1947.

It has been shown by Lipschutz and his associates that the tumorigenic action of oestrogens depends upon its *continuous* absorption (Lipschutz, 1942; see also Iglesias, 1938). The following two facts may be mentioned here. The quantity of α-oestradiol necessary to maintain the uterine weight of a castrated guinea-pig at the normal level of about 1 g. is somewhat less than 1 μg. given three times weekly by subcutaneous injections (Lipschutz, Rodríguez and y Vargas, 1939). On the other hand the quantity necessary to induce abdominal fibroids is not much above the mentioned hysterotrophic dose; abdominal fibroids can be induced by three injections per week of 5 μg. of α-oestradiol, or even less, when administered as the 17-caprylic ester of α-oestradiol, which is absorbed so slowly that a continuous oestrogenic action is made possible (Lipschutz, Bellolio, Chaume and Vargas, 1941). Similarly a few micrograms of free α-oestradiol or oestrone absorbed from a subcutaneously implanted tablet also are sufficient to induce fibroids (Lipschutz, Thibaut and Vargas, 1942). On the contrary, quantities about a hundred times greater are necessary to elicit the same tumorigenic abdominal reaction if administered by subcutaneous injections of the free hormone (Lipschutz *et al.*, 1939, 1942).

In the present work we shall deal with abdominal fibroids induced by extremely small quantities of estrogens and with the neoplastic action of these quantities on the uterine glands in experiments of long duration.

### *Fibromatogenic Action of Very Small Quantities of α-oestradiol and of Oestradiol-dipropionate.*

Tablets consisting of one part of the oestrogen and 19 parts of cholesterol Merck were prepared in the usual way by mixing, dissolving in ether, drying and mixing again. Tablets weighed 33 to 35 mg. each. After drying *in vacuo* they were implanted beneath the skin into castrated female guinea-pigs weighing 380 to 480 g. Necropsy was made 112 to 118 days later. Uterine and other abdominal fibroids were elicited under these experimental conditions (Riesco, 1942), though the incidence was less than with the injection of great quantities of oestradiol administered as the benzoic ester, as in the experiments of Iglesias (1938).

Absorption was calculated from the loss of weight of the tablets recovered during necropsy, cleaned and dried subsequently. Calculation was made under

* This work has been aided by a grant from The Jane Coffin Childs Memorial Fund for Medical Research, grant administered by Prof. A. Lipschutz.

the supposition that the concentration of oestrogen in the tablet has not changed, as has been shown in this Department by Fuenzalida (1944) for different steroids. Such a calculation is a very rough one, since it is known that absorption is at the beginning greater than later on. In our work, with only 5 per cent of oestrogen in the tablet, chemical control of concentration was impossible. A biological control of the recovered tablets was made by reimplanting tablets a second, third and fourth time in different animals (Riesco, 1944). The result of these experiments is given in Table I.

As seen from Table I, tablets containing originally 5 per cent of the free α-oestradiol and remaining for about 4 months in the body of a guinea-pig produce anew oestrogenic and fibromatogenic actions when reimplanted into another guinea-pig. The same result was observed when 4 months afterwards the tablet was implanted for a third time and a fourth time. There was in all these experiments opening of the vagina, growth of the nipples and uterine bleeding. At necropsy the uterus was greatly increased and often monstrous, weighing 17 and even 34 gr., in animals with tablets which before implantation were already active in other animals for almost a year (see tablet No. 9 and No. 10, fourth implantation). There were uterine and other abdominal fibroids in most of these animals. Fibromatogenic action did not diminish with tablets which had been already used in previous implantations.

The total quantity of oestrogen in these tablets weighing 33 to 35 mg. was 1650 to 1750 μg. Should there have been selective absorption, oestrogenic hysterotrophic and fibromatogenic actions or uterine bleeding would have been impossible, in any case, in the third and fourth implantation. When roughly calculating the average daily absorption from the loss of weight of the tablet and supposing non-selective absorption, figures ranging between 0·9 and 1·5 μg. were obtained for the first 4 months. It is remarkable that in successive implantation smaller figures were obtained. Allowance must be made for entering of substances into the tablet as has been shown by different authors (Bishop and Folley, 1944; Deanesly and Parkes, 1943). Absorption was most probably greater than indicated by figures in our Table I. But it can easily be demonstrated that daily absorption of oestradiol was *certainly less than* about *3 to 4 μg.* In these experiments the total quantity of oestradiol in the tablet was never more than 1750 μg., and the tablet remained beneath the skin during 470 days without exhaustion of the oestrogen. But in the next paragraph we shall mention experiments in which similar tablets were active for a total of *675 days without the oestrogen being exhausted.*

Histological examination of the uterus in the mentioned animals revealed pronounced cystic glandular hyperplasia of the endometrium, polypous proliferation of the latter, thickening of the vaginal wall and proliferation of mammary tissue. These statements coincide with what has been formerly observed with the prolonged action of estrogens in this Department.

*Neoplastic Action of Small Quantities of Oestrogens on the Uterine Glands in Experiments of Long Duration.*

In the above-mentioned preliminary experiments evidence was given that by continuous absorption of very small quantities of oestrogen, certainly less than 3 to 4 μg. per day, fibromatogenic and epitheliotoxic actions on the endometrium were induced. In the present paragraph results with animals will be given in which the same small quantities of the oestrogens were active for 517 to 552 days in the same animal.

Comparative experiments were made with tablets containing 5 per cent of oestradiol dipropionate and 95 per cent of cholesterol Merck. Results are given in Table II.

There were in the first experiment six castrated guinea-pigs, which were necropsied 119 to 129 days after implantation of the tablet. Results were similar to those described in the former paragraph with free α-oestradiol. Fibromatogenic action was rather less pronounced than with the free hormones. But with the fibromatogenic action of oestrogens due allowance has to be made for variation.

In the second series in which tablets already used in the first one were again implanted and left in the body for no less than one and a half years, uterine growth was considerable. Uterine fibroids were also induced.

Microscopical examination of the uterus revealed in the series of long duration a condition fundamentally different from that seen at four months ; there was besides cystic glandular hyperplasia of the endometrium also proliferation of glands infiltrating the myometrium, *reaching the serosa and even perforating it.* The lumen of the proliferating and infiltrating glands was sometimes cystic, sometimes small or absent. The nuclei of the cells of these structures seemed normal as to size and shape, but revealed hyperchromatism according to the statement of the pathologist. Contrary to the normal, the protoplasm was basophylic. There was no inflammatory reaction. The condition was similar in all the five animals examined. The pathologist, Prof. I. Mena, declared the condition to be that of an adenocarcinoma.

### DISCUSSION.

The two comparative series described in the last paragraph show conclusively that small quantities of the oestrogen, which in experiments of four months' duration produced cystic glandular proliferation, elicit a different condition if allowed to act for 18 months ; there was an invasion of proliferating glands into the myometrium. A similar picture has been described by Lipschutz (1937) in experiments in which the ovary was reduced to a small fragment. The duration of the experiments was of 30 to 33 months. The neoplastic and infiltrative growth of the glands was, in Lipschutz' experiments with ovarian fragmentation, coincident with those we have described in the last paragraph, with the exception that with ovarian fragmentation the invading glands did not reach the serosa and did not perforate it, as was the case in one of our animals. When comparing

FIG. 1.—Proliferated uterine glands of different diameter, some cystic. The glands are invading the myometrium and come very near to the uterine serosa. Animal No. 57, Table II, 552 days of oestrogenic action. × 70.

FIG. 2.—Isolated gland, almost transformed into an epithelial cord, between the circular and longitudinal part of the myometrium. Animal No. 61, Table II, 517 days of oestrogenic action. × 135.

FIG. 3.—Above, proliferated uterine glands reach the serosa which is destroyed. Below, proliferation of the endothelium of the serosa. Animal No. 56, Table II, 552 days of oestrogenic action. × 135.

FIG. 4.—Proliferated uterine glands which have penetrated the uterine serosa and have mingled with the proliferated endothelium of the serosa. The same animal as Fig. 3. × 135.

FIG. 5.—Uterine glands in the myometrium near the serosa and proliferated endothelium of the serosa at higher magnification. The same animal as Fig. 3 and 4. × 300.

1

2

4

3

5

Riesco.

results with ovarian fragmentation and the present experiments the following statement can tentatively be made: With subcutaneously implanted tablets the infiltrative growth proceeded further than the ovarian fragmentation because in the latter case follicular phases, though prolonged, were occasionally interrupted by luteal phases.

So far as known to the author, there were no statements about similar atypical growth experimentally induced by oestrogens in the uterus of the guinea-pig besides those with ovarian fragmentation mentioned above. Another statement made by Lipschutz and his associates also deserves to be mentioned here (Lipschutz, Iglesias and Vargas, 1939). A guinea-pig having been injected for 8 months with oestradiol benzoate was found, 4 months later, to have a "pre-cancerous" transformation of the endometrium in the upper third of the uterine horn. This latter observation of Lipschutz and others is so far the only one which may be quoted in favour of irreversible atypical proliferation of the uterine mucosa elicited in the guinea-pig by oestrogens and persisting after the withdrawal of the hormone.

Neoplastic malignant growth seems to be more easily elicited in the *cervix* of the uterus in the *mouse*, as shown by the work of Gardner and others (Gardner, Allen, Smith and Strong, 1938) and Allen and Gardner (1941). In the work of these authors the oestrogens—the benzoic ester of oestrone and oestradiol—were given by injection. The quantities injected were, when calculated per gram of body weight in the work of the American authors with mice, probably 20 times greater than in our work with guinea-pigs.

Another interesting aspect of our work may be mentioned here. The guinea-pig in which invasive growth of the uterine glands was obtained by ovarian fragmentation or by the prolonged action of hormones absorbed from subcutaneously implanted tablets in experiments of long duration, is highly resistant against the carcinogenic action of hydrocarbons. Lipschutz (unpublished) and other authors were unable to elicit epithelial tumours of the skin in the guinea-pig by painting with benzpyrene or methylcolanthrene. Iglesias and Riesco (unpublished) have worked with cylindric pellets of methylcholanthrene implanted subcutaneously into the abdominal cavity and into the brain of guinea-pigs; there were no tumours in animals necropsied more than 500 days later. Shimkin and Mider (1941) were more fortunate with their strain of guinea-pigs in which malignant tumours were induced by injections of methylcholanthrene. Spontaneous tumours are also very rare in guinea-pigs, as shown by the work of Lipschutz (1941), Riesco and Schwarz (1944) and others.

### SUMMARY.

Abdominal fibroids, glandular hyperplasia and adenomatous polyps were induced in 4 months in castrated female guinea-pigs with the absorption of less than 3 to 4 μg. of α-oestradiol free or esterified, per day administered by subcutaneously implanted tablets of the hormone mixed with cholesterol.

When the same, or smaller, quantities of the oestrogen were allowed to act for 18 months there was besides abdominal fibroids and cystic glandular hyperplasia also an invasive growth of the uterine glands in all the animals histologically examined.

The proliferating uterine glands which infiltrated the myometrium, reached the serosa and in one case perforated it.

Thanks are due to Prof. A. Lipschutz, to Dr. I. Mena and to Dr. Fuenzalida for helpful criticism, to Miss A. Contreras and Mr. S. Chaskel for technical help.

REFERENCES.

ALLEN, E., AND GARDNER, W. U.—(1941) *Cancer Research*, **1**, 359.
BISHOP, P. M. F., AND FOLLEY, S. J.—(1944) *Lancet*, i, 434.
DEANESLY, R., AND PARKES, A. S.—(1943) *Ibid.*, ii, 500.
FUENZALIDA, F.—(1944) *Rev. Canadienne de Biol.*, **3**, 366.
GARDNER, W. U., ALLEN, E., SMITH, G. M., AND STRONG, L. C.—(1938) *J. Amer. med. Ass.*, **110**, 1182.
IGLESIAS, R.—(1938) *Tesis Universidad de Chile* (Public. Med. Exper., No. 1).
LIPSCHUTZ, A.—(1937) *Gynéc. et Obstet.*, **36**, 407.—(1942) *Rev. Med. y Alim.* (*Chile*), **5**, 61.—(1941) *Arch. Pathol.*, **31**, 702.
*Idem*, BELLOLIO, P., CHAUME, J., AND VARGAS, L.—(1941) *Proc. Soc. exp. Biol. N.Y.*, **46**, 164.
*Idem*, IGLESIAS, R., AND VARGAS, J., Jr.—(1939) *C. R. Soc. Biol., Paris*, **130**, 1536.
*Idem* AND MAASS, M.—(1944) *Cancer Research*, **4**, 18.
*Idem*, RODRÍGUEZ, F., AND Y VARGAS, L.—(1939) *C. R. Soc. Biol., Paris*, **130**, 939.
*Idem*, THIBAUT, R., AND VARGAS, L.—(1942) *Cancer Research*, **2**, 45.
RIESCO, A.—(1942) *Rev. Med. y Alim* (*Chile*), **5**, 158.—(1944) *Bol. Soc. Biol.* (*Chile*), **1**, 35.
*Idem* AND Y SCHWARZ, J.—(1944) *Ibid.*, **1**, 61.
SHIMKIN, M., AND MIDER, B.—(1941) *J. Nat. Cancer*, **1**, 707.